# Disturbances in the IgG Antibody Profile in HIV-Exposed Uninfected Infants Associated with Maternal Factors

**DOI:** 10.1155/2024/8815767

**Published:** 2024-02-12

**Authors:** Rodrigo T. Camacho-Pacheco, Jessica Hernández-Pineda, Yesenia Brito-Pérez, Noemi Plazola-Camacho, Irma A. Coronado-Zarco, Gabriela Arreola-Ramírez, Mextli Y. Bermejo-Haro, M. Angel Najera-Hernández, Gabriela González-Pérez, Alma Herrera-Salazar, Andrea Olmos-Ortiz, Diana Soriano-Becerril, Claudia Sandoval-Montes, Ricardo Figueroa-Damian, Sandra Rodríguez-Martínez, Ismael Mancilla-Herrera

**Affiliations:** ^1^Infectology and Immunology Department, National Institute of Perinatology (INPer), Mexico City, Mexico; ^2^Departamento de Inmunología, Escuela Nacional de Ciencias Biológicas, Instituto Politécnico Nacional, Ciudad de México, Mexico; ^3^Posgrado en Inmunología, Escuela Nacional de Ciencias Biológicas, Instituto Politécnico Nacional, Mexico City, Mexico; ^4^Neonatology Department, National Institute of Perinatology (INPer), Mexico City, Mexico; ^5^Department of Physiology and Cellular Development, National Institute of Perinatology (INPer), Mexico City, Mexico; ^6^Unidad de Investigación Multidisciplinaria, Facultad de Estudios Superiores Cuautitlán, UNAM, Cuautitlán Izcalli, Mexico; ^7^Immunobiochemistry Department, National Institute of Perinatology (INPer), Mexico City, Mexico

## Abstract

Over the last 20 years, the incidence of vertical HIV transmission has decreased from 25%–42% to less than 1%. Although there are no signs of infection, the health of HIV-exposed uninfected (HEU) infants is notoriously affected during the first months of life, with opportunistic infections being the most common disease. Some studies have reported effects on the vertical transfer of antibodies, but little is known about the subclass distribution of these antibodies. We proposed to evaluate the total IgG concentration and its subclasses in HIV+ mothers and HEU pairs and to determine which maternal factors condition their levels. In this study, plasma from 69 HEU newborns, their mothers, and 71 control pairs was quantified via immunoassays for each IgG isotype. Furthermore, we followed the antibody profile of HEUs throughout the first year of life. We showed that mothers present an antibody profile characterized by high concentrations of IgG1 and IgG3 but reduced IgG2, and HEU infants are born with an IgG subclass profile similar to that of their maternal pair. Interestingly, this passively transferred profile could remain influenced even during their own antibody production in HEU infants, depending on maternal conditions such as CD4+ T-cell counts and maternal antiretroviral treatment. Our findings indicate that HEU infants exhibit an altered IgG subclass profile influenced by maternal factors, potentially contributing to their increased susceptibility to infections.

## 1. Introduction

Prevention of mother-to-child HIV transmission has become one of the greatest successes in medical history. This was made possible by the implementation of c-section delivery, breastfeeding avoidance, and prophylactic antiretroviral treatment (ART) [1], which, to date, has reduced the incidence of vertical transmission to less than 1% [2]. *In utero* HIV-exposed uninfected (HEU) infants are not HIV infected, but they spend their first 2 years of life with a three-fold higher risk of mortality [3] and higher susceptibility to infections caused by opportunistic infection [3–5] than HIV-unexposed uninfected (HUU) infants. This increased susceptibility to disease suggests perturbations of defense mechanisms.

In HEU infants, several immune alterations, including classical innate [6–9] and adaptative immune perturbations have been described, mainly in T-cell responses [10–14]. However, protection in the first months of life depends not only on the neonatal immune system but also on passive immunity transferred by mothers through antibodies [15]. In a noncomplicated term pregnancy, starting in gestational week 13 and peeking in the third trimester, a large amount of maternal IgG antibodies cross the placenta [16]. These immunoglobulins are taken from the maternal circulation by fluid-phase endocytosis in syncytiotrophoblasts. Once internalized, these immunoglobulins bind to the neonatal Fc receptor (FcRn) in endocytic vacuoles and are released to the basolateral side of the neonatal circulation [17]. Transplacental IgG transport depends on several factors, including concentration, subclass antibodies (IgG3 > IgG1 > IgG4 > IgG2), and maternal health status [18–20].

For viral infections, the humoral immune response favors Fc effector functions for the rapid clearance of pathogens [19–25], and for HIV, IgG1 and IgG3 are the abundant immunoglobulins that exert this kind of protection [26]. A polarized profile of IgG antibodies is a characteristic of certain pathogens or vaccines; for example, severe acute respiratory syndrome coronavirus 2 (SARS-CoV-2) induces an imbalanced IgG1 and IgG3 response [27], but the *Streptococcus pneumoniae* capsular polysaccharide vaccine induces IgG2 [28]. Importantly, in HIV-exposed newborns, a reduced transference of maternal-specific antibodies against several pathogens has been reported [12], but it is still unknown whether the vertically obtained IgG subclass profile is affected in these infants.

We hypothesize that the antibody profile of HIV infection can be vertically transferred to HEU newborns in a maternal health status-dependent manner that affects neonatal humoral defense, leading to increased susceptibility to infection. In this study, we evaluated the antibody class and subclass distribution of HEU infants and analyzed its relationship with maternal clinical status and treatment.

## 2. Materials and Methods

This study was evaluated by the Research, Biosecurity and Ethics Committees of the National Institute of Perinatology (INPer), Mexico City, and was funded by the 2019-1-31 and 2021-1-14 grants from the same institute.

### 2.1. Patients and Samples

HIV-1-exposed neonates born to HIV-infected mothers who attended the INPer were included. The general criteria for participants included neonates who were born at term or who were singletons and had a normal birth weight and no congenital anomalies. Complications associated with maternal comorbidities were excluded, and all mothers received ART during pregnancy. After the HEU mothers provided signed informed consent, 500 *µ*L of peripheral blood was obtained from both the mothers and the HEU neonates on the first day after birth. Furthermore, three follow-up blood samples from HEU infants were obtained at 2–3, 4−5, and 6–12 months. Umbilical cord blood samples from newborns born via uncomplicated cesarean delivery and peripheral blood from their corresponding mothers were used as controls. Moreover, the HEU group was stratified according to the predominant ART regimens prescribed to their mothers during pregnancy as follows: Group 1: darunavir + emtricitabine + abacavir + ritonavir + tenofovir; Group 2: efavirenz + emtricitabine + tenofovir; Group 3: emtricitabine + abacavir + tenofovir; and Group 4: emtricitabine + lopinavir + ritonavir + tenofovir.

### 2.2. Immunoglobulins Quantification

Plasma samples were analyzed using the LEGENDplex^TM^ Human Ig Isotyping Panel (8-plex) Multi-Analyte Flow Assay Kit (cat. 740637, BioLegend, San Diego, CA). Briefly, 200 *µ*L of blood was centrifuged at 1,000x *g* for 10 min to separate the plasma. One microliter of plasma was diluted 1 : 10,000,000 with Assay Buffer. Twenty-five microliters of diluted plasma sample were incubated for 2 hr with 25 *µ*L of capture beads. After incubation, the beads were washed with wash buffer and centrifuged at 200x *g* for 5 min. Then, 25 *µ*L of detection antibody mixture was added and incubated for 1 hr. Subsequently, the beads were washed again and incubated for 30 min with 25 *µ*L of SA-PE. A final wash was performed, and the samples were reconstituted in 200 *µ*L of wash buffer. Samples were acquired in a FACS ARIA III cytometer with DIVA V8.0.2 software (BD Biosciences). Log-transformed data were used to construct standard curves fitted to eight discrete points using a 5-parameter logistic model. Concentrations were calculated using interpolation of the corresponding standard reference curves.

### 2.3. Quantification of Protease Inhibitor (lopinavir) Levels in Plasma Samples

Lopinavir levels in plasma samples were analyzed using ultrahigh-performance liquid chromatography coupled with tandem mass spectrometry (UPLC‒MS/MS), which was developed and validated in house according to national and international guidelines [29–31]. An ACQUITY UPLC H-Class System coupled to a Xevo TQ-S tandem mass detector equipped with an ESI source from Waters (Waters Corp., Milford, MA, USA) was used. Data acquisition, peak integration, data processing, and reporting were performed with MassLynk version 4.1 software (Waters Corp.). Chromatographic separation was performed on an Acquity BEH C18 column (2.1 *µ*m × 50 mm, 1.7 *µ*m) at 35°C under gradient conditions using acetonitrile and formic acid (0.1%) as the mobile phase, with a 3-*µ*L volume injection and run-time of 3.0 min. For spectrometric mass detection, we used electrospray positive ions (ESI+) in multiple reaction monitoring (MRM) mode. The protonated precursor/product ion transitions for lopinavir were set at *m*/*z* 629.55/447.35. Concentrations were quantified in 100-*µ*L plasma samples after protein precipitation using 0.1% formic acid in cold acetonitrile using interpolation of the corresponding standard reference curve. This range was 250–15,000 lopinavir plasma standards.

### 2.4. Statistical Analyses

The differences between two groups were calculated using the Mann‒Whitney unpaired test and the Kruskal‒Wallis test with Dunn post hoc test for three or more groups. Measures of central tendency are expressed as medians with interquartile ranges. Categorical variables are presented as frequencies and were analyzed with the same group criteria. The significance of all tests was defined as *p*  < 0.05. All analyses were performed using Prism software version 9 (GraphPad, La Jolla, CA, USA).

## 3. Results

In this study, we analyzed the plasma antibody levels of 39 HIV-infected mothers, 71 control mothers, 69 HEU newborns, and 71 HUU newborns. Detailed descriptions of the mothers and infants are summarized in Table 1. Briefly, HIV+ mothers were slightly younger than control mothers and had a considerably lower percentage of CD4+ T cells, with a total mean count of 1,061 ± 377.8 cells/mL and a mean viral load of 6,954 ± 3,644 copies/mL. The gestational age, length, and weight at birth, sex, and percentage of Th cells were similar between the HEU and HUU infants. The cephalic perimeter was slightly smaller in HEU infants, and most of them were born by c-section.

### 3.1. HEU Newborns Exhibit an Altered IgG Subclass Profile

**Figure 1:**
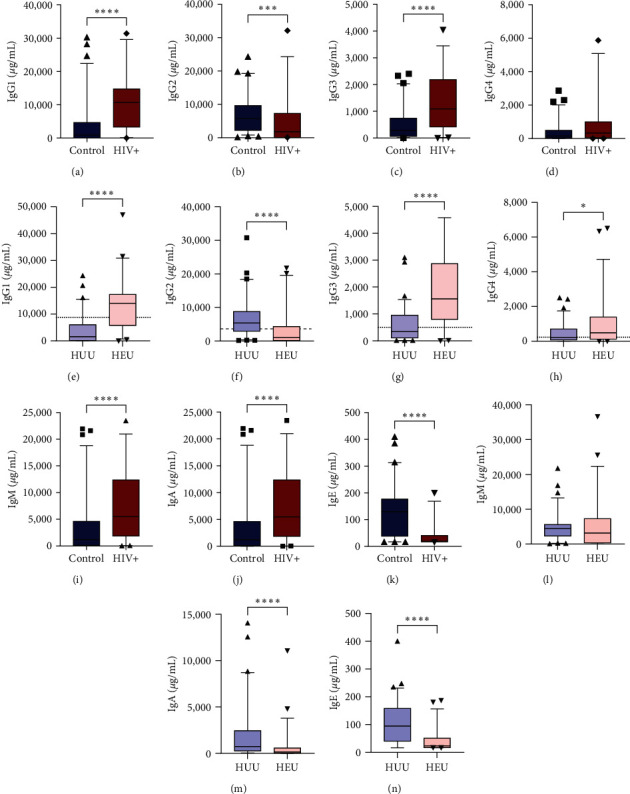
Altered IgG subclass profile in HEU newborns. The plasma concentrations of the antibodies were quantified using multianalyte flow assays. Quantification of IgG1, IgG2, IgG3, and IgG4 in mothers (a–d) and newborns (e–h). Plasmatic concentrations of IgM, IgA, and IgE in mothers (i–k) and newborns (l–n). Red denotes HIV–HEU pairs, while blue represents control mother–newborn pairs. The adult control group included 71 adults, 39 in the HIV group, 69 in the HUU group, and 54 in the HEU group. Boxes indicate medians and interquartile ranges, with bars indicating percentiles 5 and 95. The sparse data points indicate observations outside these percentiles. Significant differences between groups were assessed using Mann–Whitney unpaired tests;  ^*∗*^*p* < 0.05,  ^*∗∗∗*^*p* < 0.001, and  ^*∗∗∗∗*^*p* < 0.0001. The dotted line represents reference values reported in Schauer et al.'s [32] study.

To characterize the antibody profiles of mothers and newborns, we quantified the concentrations of antibodies in maternal and neonatal blood. As shown in Figure 1, compared with those in the control group, the profiles of IgG subclasses in the HIV-infected mothers were different (Figure 1(a)–1(d)): elevated IgG1 (median SD, 907.9 ± 6,431 vs. 10,746 ± 8,607 *μ*g/mL; *p* < 0.0001) and IgG3 (290.2 ± 567 vs. 1,090 ± 1,121 *μ*g/mL; *p* < 0.0001) but lower IgG2 (5,723 ± 5,466 vs. 1,811 ± 7,007 *μ*g/mL; *p*=0.0002) and a trend toward lower IgG4 (159.3 ± 593.6 vs. 333.9 ± 1,392 *μ*g/mL; *p*=0.1370) antibody concentrations. Interestingly, HEU newborns presented the same biased distribution of IgG subclasses as did the matching mothers (Figure 1(e)–1(h)): elevated IgG1 (1,562 ± 5,124 vs. 14,044 ± 9,191 *μ*g/mL; *p* < 0.0001) and IgG3 (338.6 ± 627 vs. 1,556 ± 1,356 *μ*g/mL; *p* < 0.0001) but lower IgG2 (5,342 ± 5,451 vs. 1,004 ± 4,935 *μ*g/mL; *p* < 0.0001) and IgG4 (205.4 ± 557.6 vs. 479.0 ± 1,451 *μ*g/mL; *p*=0.0497). The main source of neonatal IgG antibodies is maternally transferred through the placenta, but fetuses themselves produce IgG antibodies, as well as IgM, IgA, or IgE during gestation [33–35]. On this basis, the concentrations of neonatal IgM, IgA, and IgE did not show the same trend (Figure 1(l)–1(n)) as those of their mothers (Figure 1(i)–1(k)). Furthermore, notable differences were observed in HEU newborns, especially in terms of IgA and IgE concentrations, which were lower than those in control newborns (735.5 ± 3,072 vs. 156.3 ± 1,765 *μ*g/mL; *p* < 0.0001 and 94.67 ± 75.55 vs. 23.37 ± 44.31 *μ*g/mL; *p* < 0.0001, respectively). All these results showed that HEU newborns are born with an altered profile of antibody classes and IgG subclasses.

Normally, the concentrations of all maternal IgG subclasses in newborns are directly related to maternal availability [36–38]. To assess the dependence of maternal transference of IgG subclasses to HEU, we correlated the concentrations of all immunoglobulins in each pair of mothers and newborns (Table 2). Moreover, control dyads presented significant correlations in most IgG subclasses, and HIV-exposed pairs did not show significant correlations. These results indicate that the concentrations of IgG in HIV mothers are not the only factor that determines IgG concentrations in HEU newborns.

### 3.2. The Profile of IgG Subclasses Remains Unchanged Throughout the First Year of Life in HEU Infants

**Figure 2:**
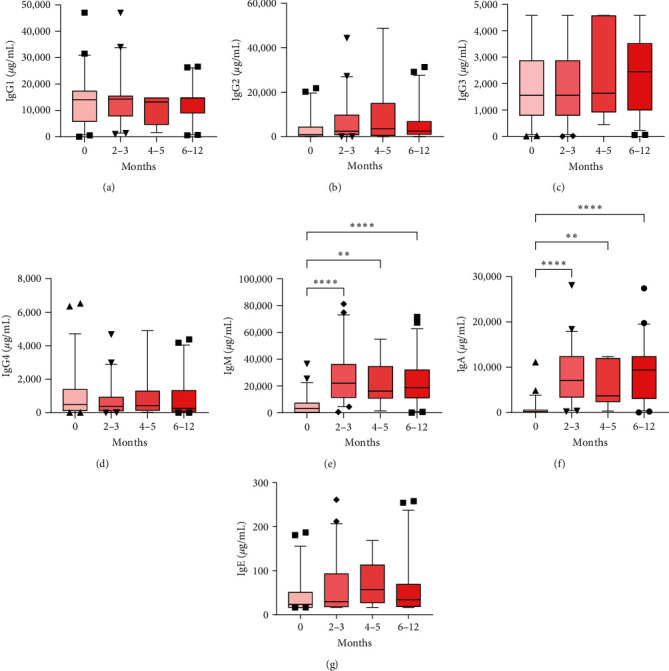
IgG antibody concentrations in HEU infants do not change during the first year of life. Plasma concentrations of IgG1 (a), IgG2 (b), IgG3 (c), IgG4 (d), IgM (e), IgA (f), and IgE (g) were quantified using multianalyte flow assays in HEU infants during their first year of life. The 0-month group comprised *n* = 54, the 2–3 months group comprised *n* = 43, the 4–5 months group comprised *n* = 9, and the 6–12 months group comprised *n* = 46. Boxes indicate medians and interquartile ranges, with bars indicating percentiles 5 and 95. The sparse data points indicate observations outside these percentiles. Significant differences between groups were assessed using the Kruskal‒Wallis test with Dunn´s post hoc test;  ^*∗∗*^*p* < 0.01, and  ^*∗∗∗∗*^*p* < 0.0001.

Ordinarily, within the first year of life, IgG antibody concentrations decline in the first 3 months due to the degradation of maternal antibodies, followed by the infant's independent production of antibodies [39, 40]. To investigate the trajectory of plasma antibody levels, we conducted quantitative assessments of immunoglobulin concentrations in HEU infants throughout their first year of life. Throughout this duration, we noted consistent concentrations of all IgG antibody subclasses from birth to 12 months (Figure 2(a)–2(d)). Interestingly, the IgM and IgA antibodies increased proportionally to the survival time (Figure 2(e)–2(g). These results indicate that HEU infants sustain the IgG antibody profile throughout the first year of life.

### 3.3. Maternal ART Influence on Infant Antibody Profiles

**Figure 3:**
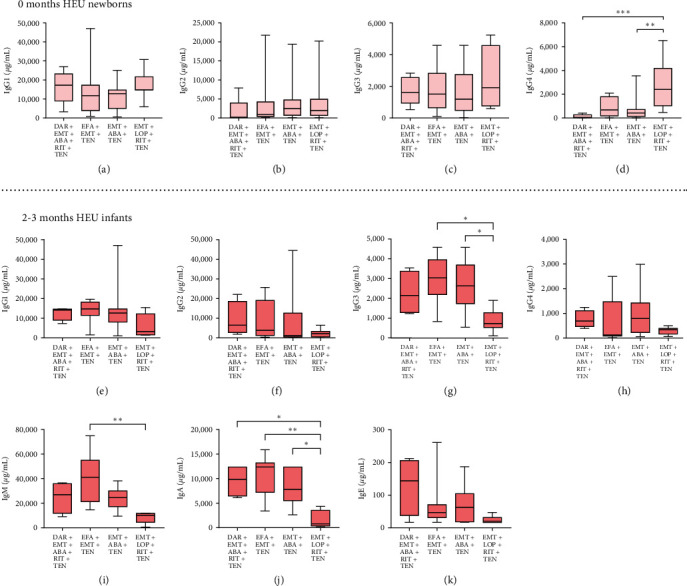
Influence of ART regimen exposure on the antibody profile of HEU infants. Plasma concentrations of IgG1 (a), IgG2 (b), IgG3 (c), and IgG4 (d) in HEU newborns divided by maternal ART regimen group. Plasma concentrations of IgG1 (e), IgG2 (f), IgG3 (g), IgG4 (h), IgM (i), IgA (j), and IgE (k) in 2- to 3-month-old HEU infants stratified according to the predominant ART regimens. The 0-month-old group comprised (group 1, *n* = 5; group 2, *n* = 15; group 3, *n* = 15; group 4, *n* = 11); and the 2- to 3-month-old group comprised (group 1, *n* = 4; group 2, *n* = 9; group 3, *n* = 12; group 4, *n* = 6). Boxes indicate medians and interquartile ranges, with bars indicating percentiles 5 and 95. The sparse data points indicate observations outside these percentiles. Significant differences between groups were assessed using the Kruskal‒Wallis test with Dunn's post hoc test;  ^*∗*^*p* < 0.05,  ^*∗∗*^*p* < 0.01, and  ^*∗∗∗*^*p* < 0.001. ABA, abacavir; DAR, darunavir; EFA, efavirenz; EMT, emtricitabina; LOP, lopinavir; RIT, ritonavir; TEN, tenofovir; and ART, antirretroviral treatment.

Several maternal factors, including viral load, CD4 count, concomitant infections, and ART, affect the immunological status of HEUs [41]. To explore the factors that condition the altered immunoglobulin profile in HEU infants, we analyzed the antibody profile of HEU infants according to maternal HIV viral load, CD4 count, concomitant infections, and ART treatments. We observed that maternal viral load and concomitant infections during pregnancy did not have an evident influence on newborn antibody concentrations. Interestingly, HEU newborns from mothers who had the highest number of CD4+ T cells had higher IgG3 and IgG4 levels (*p*=0.0421 and *p*=0.0210) (*Supplementary 1*). Interestingly, we found significant relationships between maternal exposure to ART and IgG4 concentrations at birth (*p*=0.0005) and between maternal exposure to ART and IgG3 (*p*=0.0139), IgM (*p*=0.0034), and IgA (*p*=0.0026) concentrations at 2–3 months (*Supplementary 1*) but not at 6–12 months (*Supplementary 1*). Individual analysis revealed that HEU newborns exposed to Group 4 ARTs employing emtricitabine, lopinavir, ritonavir, and tenofovir had higher IgG4 concentrations than the remaining HEU newborns exposed to other maternal treatments (Figure 3(a)–3(d)). Furthermore, the same group also had the lowest concentrations of IgG3, IgM, and IgA at 2–3 months (Figure 3(g), 3(i), 3(j)); even those immunoglobulins that did not show significant differences had very similar tendencies (Figure 3(e), 3(f), 3(h), 3(k)). In contrast to the ART regimens used in the other groups, lopinavir was used in Group 4. Hence, we hypothesized that the concentrations of this particular ART could explain the disparities observed among other regimens. Consequently, we quantified the maternal concentration of lopinavir at birth and observed negative correlations with all infant immunoglobulins at 2–3 months (*Supplementary 2*). These findings collectively indicate that maternal ART significantly influences the profile of newborn antibodies.

Additionally, we analyzed whether newborn factors such as gestational age, birth weight, birth length, and birth CD4 count influenced the immunoglobin concentration at any point in the first year of life in the HEU infants, but no important relationships were found (*Supplementary 1*). All these results suggest that the HEU newborn antibody concentration is heavily influenced by the maternal environment, as characterized by CD4+ T-cell counts or ART.

## 4. Discussion

Growing evidence has highlighted the significance of the maternal environment for neonatal and infant defense mechanisms in children that are born to HIV+ mothers but not infected [41]. Both epidemiological and experimental data indicate potential impairment in the humoral responses of HEU infants. This study found that HEU infants are born with a modified IgG subclass profile, which is influenced by maternal factors such as CD4+ T-cell counts and ART. Interestingly, this passively transferred profile could remain affected even when HEU infants produce IgG antibodies themselves.

For viral responses, the immune system employs a specialized profile of humoral mediators favored by IgG1 and IgG3 antibodies [19, 24, 25], which are focused on neutralization, opsonization, Fc effector functions (inflammation and antibody-dependent cell-mediated cytotoxicity (ADCC)), intracellular virus degradation, and complement activation for rapid clearance of pathogens [21–23]. Previous studies have reported an unbalanced subclass profile characterized by elevated IgG1 and IgG3 but reduced IgG2 and IgG4, the main antibody response to HIV infection [26]. Consistent with these findings, we report increased concentrations of total IgG1 and IgG3 and reduced concentrations of IgG2 in pregnant mothers with HIV infection (Figure 1(e)–1(g)), which are independent of the normal increase in the IgG1 and IgG3 subclasses caused by gestation [42]. Furthermore, the maternal repertoire of antibodies represents part of the immunological experience of the mother, and the transfer of antibodies from the placenta to the fetus provides protection against the pathogen for newborns after birth [17]. As expected, passively transferred antibodies that occur in the neonatal circulation reflect the maternal antibody profile (Figure 1(a)–1(h)), with IgG1 and IgG3 being the main IgG subclasses of immunoglobulins in HEU newborns. Several studies have shown that total specific IgGs from HEU infants exhibit decreased surface binding and antibody-mediated C3 b/iC3b deposition [43], which corresponds to the function and predominance of the subclasses; most of these subclasses could be IgG1 and IgG3 according to our results. Despite the enrichment of other IgG subclasses, the levels of IgG2 in both the HEU newborns and their paired mothers were lower than those in the control group. This antibody subclass exhibits a limited capacity to engage effector functions compared to that of other IgG subclasses. However, this antibody subclass primarily contributes to immune responses against bacterial capsular polysaccharides, serving as an antibody with preferential reactivity to glycans [23, 44]. Therefore, while the IgG1 and IgG3 subclasses demonstrate increased levels, despite their limited pathogen-killing efficacy, their combination with diminished quantities of IgG2 could be partially responsible for the increased susceptibility to infections in HEU infants during the first months of life [4, 5, 45, 46].

On the other hand, neonatal IgG concentrations depend mainly on maternal IgG concentrations, and in uncomplicated pregnancies, there is a statistically direct relationship between the two compartments [15, 36, 37]. In our cohort, while the control group supported the last pattern, the HIV+ mothers-HEU newborn pairs did not follow this correlation for any IgG subclasses (Table 2). Several factors could be related to these concentration differences between immunoglobulins, including the transport conditions from maternal to fetal blood compartments and the individual fetal production of immunoglobulins. First, IgG antibodies cross the placental barrier depending on the subclass affinity for FcRn, maternal concentration, and posttranslational modifications of immunoglobulins. In terms of affinities, IgG1 and IgG3 have the strongest affinity for the receptor and are transported more than IgG2 [17, 24], thus favoring IgG1 and IgG3. In addition, high levels of total and specific IgG subclasses can saturate FcRn, altering the amount of IgG transmitted [17]. According to the above findings, IgG2 antibodies are competitively antagonized by IgG1 and IgG3. Additionally, the affinity of IgG for FcRn depends on the glycosylation pattern [47]. The environment caused by HIV infection promotes changes in the glycan arrangement of IgG antibodies, which convert them to an inflammatory phenotype of immunoglobulins. This modification adjusts effector functions and transport between compartments, including IgG transfer from the mother to the fetus [47]. In a cohort similar to the one used for these results, previous studies have reported alterations in the vertical transfer of IgG, partially associated with changes in the glycosylation pattern of antibodies rather than the expression of FcRn in the placenta [48].

Regarding the fetal production of IgG antibodies mentioned earlier, contrary to the notion that the prenatal condition is immunologically immature, some studies indicate the presence of transitional follicular B cells and memory T cells in the fetus's mucosa beginning in the second trimester of gestation [49, 50], suggesting that fetal lymphocytes are able to respond to antigenic challenges during gestation, mainly those opsonized antigens that are transported through the placenta, without discarding the immune complex observed in HIV infection [51–53]. In addition, the IgG profile could not be the only B response affected in newborns with HEU. Our results additionally demonstrated decreased IgA and IgE concentrations in HEU newborns (Figures 1(j) and 1(k). These immunoglobulins are not vertically transferred during gestation, and they are produced in neonatal blood during gestation [54, 55]. The low amounts observed might originate from preferential fetal production of IgG, particularly IgG1 and IgG3, influenced by the gestational inflammatory milieu resulting from maternal infection. However, this assumption requires further in-depth exploration.

All this evidence suggests that maternal HIV infection induces an early humoral response characterized by a predisposed IgG antibody profile favoring the IgG1 and IgG3 subclasses. It is expected that IgG concentrations decrease between the third and sixth months and then recover during this period due to the normal degradation of maternal antibodies and the infant's own production of these antibodies [39, 40]. Interestingly, the concentrations of IgG subclasses in plasma from HEU patients did not significantly change during the first year of life (Figure 2). However, IgM, IgA, and IgE did not appear to be affected during this period, leading to an expected increase (Figure 2). According to the DOHaD (Developmental Origins of Health and Disease) hypothesis, environmental factors exposed during pregnancy generate early fetal physiological adaptations to prepare offspring for future challenges [56]. In this regard, similar altered adaptations can be observed in the vaccine response of HEU infants. Following antigen-specific stimulation, peripheral blood mononuclear cells from HEU infants vaccinated with Bacillus Calmette–Guérin and tetanus exhibit a reduced frequency of proliferating T cells and a decreased occurrence of Th cells expressing effector cytokines [57–60]. Hence, we propose that HEU infants demonstrate modified programming for antibody production in IgG subclasses, which appears to persist during the first months of life without impacting other antibody classes. Further studies are needed to substantiate this hypothesis. Currently, our laboratory group is actively involved in conducting this research.

According to the results above, newborns are conditioned by their own maternal health status [61]. In this regard, the viral load and CD4 count are relevant health parameters not only for HIV patients but also for pregnancy progression [62]. It is well-documented that a decrease in CD4+ T cells, in addition to the high viral load of HIV [63], favors inflammatory responses that induce alterations in the permeability of the placenta [64, 65]. We did not observe any relationship between maternal CD4 count and viral load; rather, maternal CD4 count was positively correlated with neonatal IgG3 and IgG4 concentrations. Therefore, the lower the number of helper T cells is, the lower the concentration of these immunoglobulins, probably due to a poor maternal B response to differentiation, which in turn predisposes HEU infants to a higher incidence of infection and hospitalization than mothers with low CD4 counts [41].

Another important maternal health parameter is the presence of concomitant infections, mainly chronic infectious diseases [66]. It is plausible that if maternal HIV affects the development of the newborn immune system, other maternal infections could affect the fetus in a similar way. However, our results did not support this idea; in this analysis, we evaluated the additional effect of acute infections (human papillomavirus and ureaplasma), and HIV could conceal any additional effect.

To control maternal HIV infection and minimize the risk of vertical transmission, ART is the ideal treatment, and its active forms not only have effects on the mother but also on the fetus [67]. When we evaluated the relationship between particular combinations of ARTs and neonatal antibody concentrations, we found that lopinavir, an HIV protease inhibitor, promoted IgG4 but lowered IgG3, IgM, and IgA levels. It is not clear how this ART could affect the production of newborn antibodies. For general long-term ART, adverse effects such as hepatic failure, neuropsychosis, and hematopoietic alterations have been reported, among others [68]. Additionally, transgenerational effects have been reported since exposure to ART has been related to preterm birth, low weight, and size at birth, as well as dyslipidemia and lipodystrophy [69, 70]. Specifically, lopinavir has been associated with nonspecific inflammatory events [71], and in combination with ritonavir, it has been shown to be associated with impaired differentiation, lipid content, mitochondrial function, ROS production, and insulin sensitivity in adipocytes [72]. However, additional mechanistic research is needed to understand the possible adverse effects of lopinavir exposure *in utero*.

While some maternal conditions could be linked to the alterations observed in the antibody defense of HEU infants, not all variances could be explained by the assessed parameters. Therefore, to comprehend the impacts on HEU infants, including vaccinations and social factors, additional parameters need to be explored. Moreover, this research encountered methodological challenges. A critical concern was the absence of optimal controls due to the use of peripheral blood for HEU newborn samples and cord blood for HUU newborn samples. Nonetheless, evidence suggests comparability between peripheral and cord blood at birth in terms of proportions and functionality of immune components [73, 74]; it is desirable to have matched samples. Additionally, we present a study with a small sample size, but the relatively low incidence of this disease in Mexico restricts the possibility of obtaining a larger sample.

## 5. Conclusions

Our study demonstrated that HEU infants exhibit an antibody profile for classical viral responses mediated by IgG1 and IgG3, which is not solely determined by the HIV+ mother's antibody concentration. Our findings suggest that various factors, primarily influenced by HIV exposure and including the mother's CD4 count and ART regimen, impact both the transfer of antibodies across the placenta and likely the fetus's independent antibody production.

## Figures and Tables

**Table 1:** Demographic characteristics of HIV-unexposed uninfected (HUU) or HIV-exposed uninfected (HEU) newborns and their corresponding mothers.

	Control mothers	HIV+ mothers	*p* Values
Maternal age (years, mean ± SD)	31.4 (5.3)	26.7 (6.1)	0.0028^#^
Percentages of Th cells (mean ± SD)	45.46 (6.4)	29.4 (13.8)	<0.0001^#^
CD4 count (cells/mm^3^, mean ± SD)	n/a	1,061 (377.8)	n/a
Viral load (copies/mL: *n* (%))	n/a	6,954 (3643.9)	n/a

	HUU newborns	HEU newborns	*p* Values

Gestational age (weeks, mean ± SD)	38.3 (1.3)	37.8 (1.7)	0.0533^#^
Length at birth (cm, mean ± SD)	48.4 (2.2)	48.2 (2.2)	0.5160^#^
Birth weight (g, mean ± SD)	2,979 (374.9)	2,815 (420.1)	0.0619^#^
Cephalic perimeter (cm, mean ± SD)	34.3 (1.3)	33.6 (1.5)	**0.0423** ^**#**^
Delivery type	*N* = 4, *C* = 67	*N* = 17, *C* = 52	**<0.0001** ^**##**^
Gender	*F* = 36 *M* = 35	*F* = 35 *M* = 34	0.9973^##^
Percentages of Th cells^*π*^ (mean ± SD)	66.01 (8.7)	65.9 (11.8)	0.9429^##^
CD4 count (cells/mm^3^, mean ± SD)	n/a	2,214 (943.3)	n/a

The results are shown as the mean and standard deviation. *π* refers to the percentage of Th cells among T cells. *p* Values for differences between groups were calculated using an unpaired *t*-test (^#^) or chi-square test (^##^). Bold values represent statistically significant results. n/a, not applicable.

**Table 2:** Correlations between newborn and maternal IgG antibody concentrations.

	HUU mother–HUU newborn pairs		HIV+ mother–HEU newborn pairs	
	Spearman R	*p* Value	Spearman R	*p* Value
IgG1	0.4253	**0.0003**	0.2656	0.2576
IgG2	0.2292	0.0581	0.2527	0.2824
IgG3	0.4344	**0.0002**	0.0421	0.8600
IgG4	0.3454	**0.0037**	0.1963	0.4068

Unexposed pairs, *n* = 69; HIV-exposed pairs, *n* = 39. The results are shown with *p* and *R* values for the Spearman correlation test. Bold values represent statistically significant results.

## Data Availability

The data supporting the findings of this study are available upon request from the corresponding author.

## Supplementary Materials

Supplementary 1 Table S1: effect of maternal factors on the HEU newborn´s antibody concentration. Table S2: effect of maternal factors on the HEU 2−3-month-old infant´s antibody concentration. Table S3: effect of maternal factors on the HEU antibody concentration in 6- to 12-month-old infants. Table S4: effect of newborn health parameters on the HEU infant´s antibody concentration.

Supplementary 2 Lopinavir maternal concentrations inversely correlate with HEU infant antibody concentrations.
