# Supplementary material for: Disturbances in the IgG Antibody Profile in HIV-Exposed Uninfected Infants Associated with Maternal Factors
Source: J Immunol Res. 2024 Feb 12;2024:8815767. doi: 10.1155/2024/8815767 (PMC10876311; doi:10.1155/2024/8815767)
Supplement: Supplementary 1 — Table S1: effect of maternal factors on the HEU newborn´s antibody concentration. Table S2: effect of maternal factors on the HEU 2−3-month-old infant´s antibody concentration. Table S3: effect of maternal factors on the HEU antibody concentration in 6- to 12-month-old infants. Table S4: effect of newborn health parameters on the HEU infant´s antibody concentration. [file 8815767.f1.docx]

**Supplementary Table S1.-** Effect of maternal factors on the HEU newborn´s antibody concentration.

| Parameter |  | | **IgG1 (μg/mL)** | | **IgG2 (μg/mL)** | | **IgG3 (μg/mL)** | | **IgG4 (μg/mL)** | | **IgM (μg/mL)** | **IgA (μg/mL)** | **IgE (μg/mL)** |
| --- | --- | --- | --- | --- | --- | --- | --- | --- | --- | --- | --- | --- | --- |
| Maternal Viral Load | Undetectable | | 14429 (8.733-47069) | | 1140 (63.76-7903) | | 1613 (1.552-4581) | | 1430 (0.9160-52126) | | 3192 (31.28-42169) | 194.6 (8.513-12360) | 24.12 (16.52-187.1) |
|  | < 1000 copies/μL | | 14490 (516.3-30713) | | 935.5 (63.76-7903) | | 1276 (19.52-2987) | | 508.1 (3.184-23664) | | 2712 (31.28-9032) | 137.9 (8.513-245) | 23.42 (16.91-51.1) |
|  | > 1000 copies/μL | | 19787 (2195-31512) | | 790.8 (63.76-2446) | | 2590 (118.2-3549) | | 616.3 (20.06-20822) | | 3661 (31.28-7698) | 10.8 (8.513-181.4) | 22.81 (16.91-26.34) |
|  | P | | n.s. | | n.s. | | n.s. | | n.s. | | n.s. | n.s. | n.s. |
| Maternal CD4 count | > 500 cells/mL | | 14763 (516.3-47069) | | 1311 (63.76-20230) | | 3028 (19.52-4581) | | 671.9 (3.184-6516) | | 5216 (31.28-21286) | 439.6 (8.513-3460) | 47.6 (16.91-148) |
|  | 200-500 cells/mL | | 12085 (816.4-30713) | | 1262 (63.76-21790) | | 890.8 (93.22-4581) | | 545.1 (0.9160-6343) | | 2179 (31.28-42169) | 73.55 (8.513-12360) | 22.3 (16.52-139.3) |
|  | < 200 cells/mL | | 13784 (8.733-26980) | | 220.2 (63.76-7903) | | 1948 (1.552-4581) | | 84.34 (0.9160-1031) | | 1583 (56.47-25563) | 113.1 (8.513-1997) | 17.7 (16.91-55.36) |
|  | P | | n.s. | | n.s. | | **0.0421** | | **0.0210** | | n.s. | n.s. | n.s. |
| Maternal concomitant infections | Non | | 14378 (904.8-31512) | | 795.2 (63.76-21790) | | 1850 (93.22-4581) | | 750.7 (0.916-2603) | | 2579 (31.28-10276) | 113.1 (8.513-11070) | 20.63 (16.91-187.1) |
|  | Ureaplasma | | 13767 (3322-47069) | | 4181 (63.76-7962) | | 1610 (269.4-4581) | | 693.1 (7.126-6516) | | 3533 (31.28-10276) | 152.4 (8.513-2298) | 29.33 (16.52-148) |
|  | HPV | | 14763 (8.733-22770) | | 990.4 (63.76-20230) | | 2277 (1.552-4581) | | 413.2 (0.916-6343) | | 5216 (31.28-42169) | 439.6 (8.513-12360) | 33.46 (16.91-139.3) |
|  | Ureaplasma  + HPV | | 12136 (5410-16985) | | 1912 (589.1-5244) | | 1698 (737.6-4581) | | 516.3 (127.5-1160) | | 1711 (235.4-25563) | 13.03 (8.565-1997) | 28.77 (16.52-5536) |
|  | P | | n.s. | | n.s. | | n.s. | | n.s. | | n.s. | n.s. | n.s. |
| Maternal antirretroviral treatment | DAR + EMT + ABA + RIT + TEN | | 17257 (3209-26980) | | 98.66 (63.76-7903) | | 1619 (525.6-2829) | | 41.2 (4.664-413.2) | | 1112 (119.1-9032) | 211.9 (12.5-894.6) | 18.4 (16.91-51.1) |
|  | EFA + EMT + TEN | | 11723 (816.4-47069) | | 955.2 (63.76-21790) | | 1507 (93.22-4581) | | 671.9 (0.9160-2086) | | 2179 (31.28-36648) | 45.19 (8.513-11070) | 40.88 (16.67-180.9) |
|  | EMT + ABA + TEN | | 12805 (516.3-25053) | | 2446 (63.76-19433) | | 1189 (19.52-4581) | | 419.8 (3.184-3548) | | 6324 (31.28-25563) | 196.6 (8.513-3296) | 23.37 (16.52-133.6) |
|  | EMT + LOP + RIT + TEN | | 14763 (5847-30713) | | 1926 (63.76-20230) | | 1912 (589.1-5244) | | 2404 (470.4-6516) | | 4092 (375.7-9918) | 324.3 (80513-3460) | 29.56 (16.91-187.1) |
|  | P | n.s. | | n.s. | | n.s. | | **0.0005** | | n.s. | | n.s. | n.s. |

The results are shown as the median min–max. P values for differences between groups were calculated using the Kruskal‒Wallis test. *n.s.: Non-significant; HPV: Human papilloma virus; ABA: Abacavir; DAR: Darunavir; EFA: Efavirenz; EMT: Emtricitabina; LOP: Lopinavir; RIT: Ritonavir; TEN: Tenofovir*

**Supplementary Table S2.-** Effect of maternal factors on the HEU 2-3-month-old infant´s antibody concentration.

| Parameter |  | **IgG1 (μg/mL)** | **IgG2 (μg/mL)** | **IgG3 (μg/mL)** | **IgG4 (μg/mL)** | **IgM (μg/mL)** | **IgA (μg/mL,)** | **IgE (μg/mL)** |
| --- | --- | --- | --- | --- | --- | --- | --- | --- |
| Maternal Viral Load | Undetectable | 13735 (1040-29421) | 2812 (63.76-44462) | 2094 (112.3-4581) | 377.5 (5.2-4682) | 21782 (492-74840) | 6419 (220.2-18432) | 31.43 (16.52-261.4) |
|  | < 1000 copies/μL | 47069 (47069-47069) | 85.9 (85.9-85.9) | 2893 (2893-2893) | 58.73 (58.73-58.73) | 9532 (9532-9532) | 5406 (5406-5406) | 19.54 (19.54-19.54) |
|  | > 1000 copies/μL | 14763 (14763-32463) | 5386 (1292-9734) | 4581 (3549-4581) | 922.8 (813-1091) | 30528 (22950-81248) | 12360 (10750-28140) | 18.7 (18.4-30.11) |
|  | P | n.s. | n.s. | n.s. | n.s. | n.s. | n.s. | n.s. |
| Maternal CD4 count | > 500 cells/mL | 14763 (1040-34072) | 2327 (63.76-25692) | 1792 (112.3-4581) | 275.6 (45.78-4682) | 23938 (492-74840) | 6341 (403.3-18432) | 29.91 (16.52-261.4) |
|  | 200-500 cells/mL | 13791 (1573-27965) | 2076 (63.8-25723) | 2476 (675-4371) | 341.7 (5.2-2373) | 21529 (4415-43803) | 5788 (788.6-15868) | 23.38 (16.52-187.1) |
|  | < 200 cells/mL | 12493 (6977-14763) | 6531 (1906-44462) | 2352 (1236-4581) | 810.5 (364-2990) | 23932 (9060-29076) | 12360 (6132-12360) | 97.06 (16.91-212.2) |
|  | P | n.s. | n.s. | n.s. | n.s. | n.s. | n.s. | n.s. |
| Maternal concomitant infections | Non | 14763 (1573-32463) | 2076 (63.76-44462) | 3035 (829.6-4581) | 275.6 (19.54-2990) | 23964 (4970-81248) | 8406 (3268-28140) | 31.21 (16.91-153.9) |
|  | Ureaplasma | 11841 (7103-34072) | 1759 (108.2-25692) | 1727 (788.8-3523) | 233.7 (93.6-4682) | 22324 (5858-66231) | 4318 (783.9-12360) | 31.34 (16.52-261.4) |
|  | HPV | 13931 (1536-47069) | 3702 (63.8-15363) | 2396 (112.3-4581) | 377.5 (5.2-1443) | 11649 (492-36087) | 5769 (403.3-18432) | 18.97 (16.52-212.2) |
|  | Ureaplasma  + HPV | 6977 (6977-6977) | 27290 (27290-27290) | 1924 (1924-1924) | 1997 (1997-1997) | 24915 (24915-24915) | 12360 (12360-12360) | 100.9 (100.9-100.9) |
|  | P | n.s. | n.s. | n.s. | n.s. | n.s. | n.s. | n.s. |
| Maternal antirretroviral treatment | DAR + EMT + ABA + RIT + TEN | 14190 (7298-14763) | 6417 (1906-22166) | 2147 (1236-3537) | 691.5 (394-1246) | 26902 (9060-36492) | 9859 (6132-12360) | 144 (16.91-212.2) |
|  | EFA + EMT + TEN | 14763 (1573-19641) | 3829 (336.7-25723) | 3035 (829.6-4581) | 121.5 (19.54-2497) | 41117 (14582-74840) | 12360 (3391-15868) | 46.96 (16.91-261.4) |
|  | EMT + ABA + TEN | 12685 (1040-47069) | 1241 (85.9-44462) | 2628 (547.5-4581) | 787.9 (58.73-2990) | 24426 (9532-38136) | 7805 (2626-12360) | 62.98 (16.91-187.1) |
|  | EMT + LOP + RIT + TEN | 3245 (1408-15420) | 2086 (67.26-6498) | 731.9 (112.3-1898) | 339.1 (64.09-500.4) | 10102 (492-11969) | 786.3 (220.2-4343) | 20.89 (16.52-46.96) |
|  | P | n.s. | n.s. | **0.0139** | n.s. | **0.0034** | **0.0026** | n.s. |

The results are shown as the median min–max. P values for differences between groups were calculated using the Kruskal‒Wallis test. *n.s.: Non-significant; HPV: Human papilloma virus; ABA: Abacavir; DAR: Darunavir; EFA: Efavirenz; EMT: Emtricitabina; LOP: Lopinavir; RIT: Ritonavir; TEN: Tenofovir*.

**Supplementary Table S3.-** Effect of maternal factors on the HEU antibody concentration in 6 to 12-month-old infants.

| Parameter |  | **IgG1 (μg/mL)** | **IgG2 (μg/mL)** | **IgG3 (μg/mL)** | **IgG4 (μg/mL)** | **IgM (μg/mL)** | **IgA (μg/mL)** | **IgE (μg/mL)** |
| --- | --- | --- | --- | --- | --- | --- | --- | --- |
| Maternal Viral Load | Undetectable | 14763 (560.8-26605) | 2334 (63.8-31313) | 2655 (57.5-4581) | 279.1 (0.9-4377) | 20193 (40-71638) | 11011 (15.8-19743) | 35.2 (16.9-258) |
|  | < 1000 copies/μL | 18686 (8257-24653) | 7282 (1749-48812) | 3313 (1643-4581) | 851.1 (17.01-4907) | 31897 (11052-54944) | 12571 (3560-27424) | 48.12 (26.85-197.7) |
|  | > 1000 copies/μL | 9268 (1830-17584) | 618.6 (63.76-16846) | 1168 (936.2-2839) | 107.3 (51.63-2087) | 16530 (1126-20007) | 4564 (196.7-12360) | 35.28 (16.91-90.65) |
|  | P | n.s. | n.s. | n.s. | n.s. | n.s. | n.s. | n.s. |
| Maternal CD4 count | > 500 cells/mL | 14763 (1243-25797) | 6709 (97.93-48812) | 2671 (58.89-4581) | 262.9 (65.74-4907) | 27991 (570-54374) | 9952 (1279-19149) | 57.53 (18.4-193.3) |
|  | 200-500 cells/mL | 12780 (560.8-26605) | 2334 (63.76-20466) | 1351 (57.5-4581) | 233.8 (0.9-2790) | 16878 (39.98-54054) | 7075 (15.8-19743) | 29.91 (16.9-258) |
|  | < 200 cells/mL | 14763 (8257-22609) | 3219 (1749-24661) | 3301 (1563-4581) | 724.4 (17.01-3776) | 25182 (7627-59782) | 11686 (3560-12781) | 33.69 (18.4-254.6) |
|  | P | n.s. | n.s. | n.s. | n.s. | n.s. | n.s. | n.s. |
| Maternal concomitant infections | Non | 14763 (560.8-26286) | 2334 (63.8-31313) | 1998 (57.5-4581) | 262.9 (0.9-4377) | 16530 (40-71638) | 9952 (15.8-19743) | 35.28 (16.9-258) |
|  | Ureaplasma | 14763 (1243-19132) | 5582 (97.93-18345) | 2720 (58.89-4581) | 1378 (53.43-4178) | 24773 (570-38653) | 12360 (1279-19149) | 49.01 (29.91-193.3) |
|  | HPV | 13430 (691.6-26605) | 1903 (63.76-48812) | 2449 (630.2-4581) | 247.4 (5.4-4907) | 18394 (1126-54944) | 9897 (196.7-27424) | 27.76 (16.9-197.7) |
|  | Ureaplasma  + HPV | 14763 (14763-14763) | 4345 (4345-4345) | 3504 (3504-3504) | 1309 (1309-1309) | 35361 (35361-35361) | 5359 (5359-5359) | 18.4 (18.4-18.4) |
|  | P | n.s. | n.s. | n.s. | n.s. | n.s.- | n.s. | n.s. |
| Maternal antirretroviral treatment | DAR + EMT + ABA + RIT + TEN | 14763 (560.8-18143) | 1866 (1749-2094) | 3151 (2243-4581) | 39.15 (17.01-1131) | 18605 (18028-67408) | 12360 (11011-12781) | 26.85 (18.61-66.55) |
|  | EFA + EMT + TEN | 14763 (560.8-18143) | 1175 (63.8-31313) | 1843 (57.5-3946) | 161.3 (0.9-2790) | 9922 (39.98-35698) | 8273 (15.8-12360) | 32.55 (16.9-258) |
|  | EMT + ABA + TEN | 14763 (1436-26286) | 5582 (415.9-48812) | 2839 (904.1-4581) | 901.9 (94.14-4907) | 29604 (7325-54944) | 12360 (642.9-27424) | 35.28 (18.4-197.7) |
|  | EMT + LOP + RIT + TEN | 11842 (874.8-25797) | 5398 (63.8-29142) | 1072 (551.2-4581) | 238 (53.43-4377) | 20193 (9322-71638) | 8782 (1111-12360) | 35.28 (18.4-174.8) |
|  | P | n.s. | n.s. | n.s. | n.s. | n.s. | n.s. | n.s. |

The results are shown as the median min–max. P values for differences between groups were calculated using the Kruskal‒Wallis test. *n.s.: Non-significant; HPV: Human papilloma virus; ABA: Abacavir; DAR: Darunavir; EFA: Efavirenz; EMT: Emtricitabina; LOP: Lopinavir; RIT: Ritonavir; TEN: Tenofovir*

**Supplementary Table S4.-** Effect of newborn health parameters on the HEU infant´s antibody concentration

|  |  | Gestational age | | Birth weight | | Birth length | | Birth CD4 count | |
| --- | --- | --- | --- | --- | --- | --- | --- | --- | --- |
| Infant age group |  | R | P | R | P | R | P | R | P |
| 0  months | IgG1 | 0.136 | 0.328 | 0.048 | 0.730 | 0.109 | 0.435 | -0.072 | 0.605 |
|  | IgG2 | 0.065 | 0.638 | 0.150 | 0.278 | 0.031 | 0.822 | 0.134 | 0.333 |
|  | IgG3 | 0.309 | **0.023** | 0.163 | 0.239 | 0.041 | 0.766 | 0.129 | 0.352 |
|  | IgG4 | 0.238 | 0.083 | 0.154 | 0.266 | -0.004 | 0.979 | -0.125 | 0.366 |
|  | IgM | 0.144 | 0.299 | 0.206 | 0.135 | -0.063 | 0.649 | 0.082 | 0.556 |
|  | IgA | 0.147 | 0.288 | 0.032 | 0.820 | -0.039 | 0.779 | 0.163 | 0.240 |
|  | IgE | 0.199 | 0.148 | 0.161 | 0.246 | -0.046 | 0.743 | 0.290 | **0.034** |
| 2-3 months | IgG1 | 0.007 | 0.965 | -0.100 | 0.533 | 0.178 | 0.264 | 0.027 | 0.868 |
|  | IgG2 | 0.020 | 0.903 | 0.045 | 0.778 | 0.091 | 0.573 | 0.083 | 0.610 |
|  | IgG3 | -0.090 | 0.583 | -0.220 | 0.166 | 0.076 | 0.635 | -0.030 | 0.856 |
|  | IgG4 | 0.235 | 0.144 | 0.087 | 0.590 | 0.147 | 0.358 | -0.003 | 0.983 |
|  | IgM | -0.101 | 0.537 | -0.221 | 0.165 | 0.022 | 0.894 | 0.166 | 0.584 |
|  | IgA | -0.096 | 0.556 | -0.243 | 0.125 | -0.039 | 0.809 | 0.089 | 0.997 |
|  | IgE | -0.149 | 0.359 | 0.006 | 0.970 | -0.135 | 0.400 | -0.003 | 0.699 |
| 6-12 months | IgG1 | 0.103 | 0.498 | 0.184 | 0.217 | -0.039 | 0.797 | 0.168 | 0.259 |
|  | IgG2 | 0.075 | 0.621 | 0.084 | 0.575 | 0.194 | 0.191 | -0.150 | 0.316 |
|  | IgG3 | -0.001 | 0.997 | 0.096 | 0.522 | -0.021 | 0.891 | 0.149 | 0.319 |
|  | IgG4 | 0.254 | 0.088 | -0.035 | 0.815 | 0.184 | 0.215 | -0.146 | 0.328 |
|  | IgM | 0.150 | 0.321 | 0.274 | 0.062 | 0.213 | 0.150 | 0.056 | 0.708 |
|  | IgA | 0.041 | 0.788 | 0.046 | 0.759 | -0.099 | 0.508 | -0.036 | 0.808 |
|  | IgE | -0.172 | 0.253 | 0.034 | 0.822 | -0.059 | 0.693 | 0.030 | 0.843 |

The results are shown with P and R values for the Spearman correlation test.
