# Supplementary material for: Disturbances in the IgG Antibody Profile in HIV-Exposed Uninfected Infants Associated with Maternal Factors
Source: J Immunol Res. 2024 Feb 12;2024:8815767. doi: 10.1155/2024/8815767 (PMC10876311; doi:10.1155/2024/8815767)
Supplement: Supplementary 2 — Lopinavir maternal concentrations inversely correlate with HEU infant antibody concentrations. [file 8815767.f2.docx]

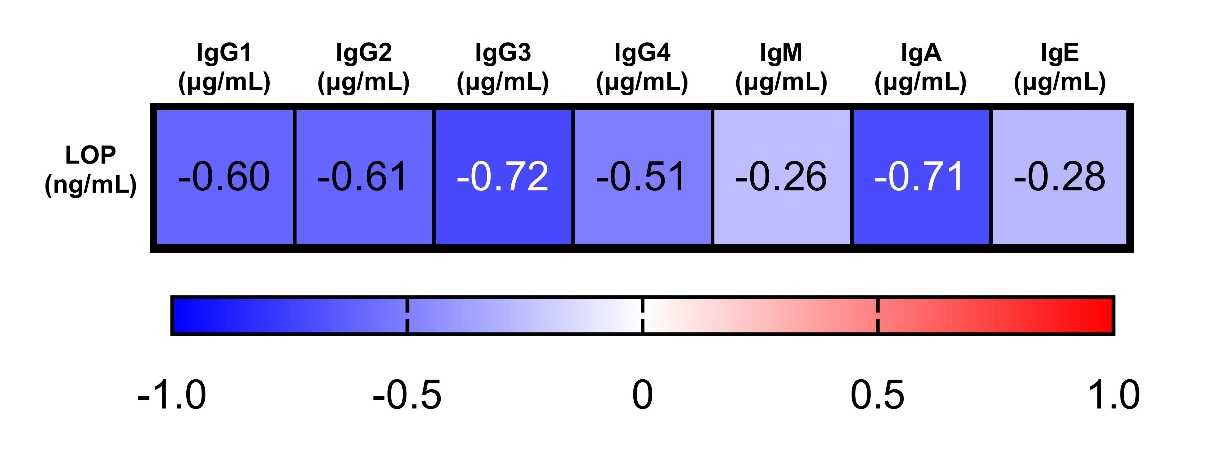


**Supplementary Figure S1.- Lopinavir maternal concentrations inversely correlate with HEU infant antibody concentrations.** Spearman correlations between maternal plasma concentrations of lopinavir at the end of pregnancy and 2-3 month-old HEU infant’s plasmatic antibody concentrations. *The results are shown with R values for the Spearman correlation test.*
